# Enrichment and Purification of Total Ginkgo Flavonoid *O*-Glycosides from Ginkgo Biloba Extract with Macroporous Resin and Evaluation of Anti-Inflammation Activities In Vitro

**DOI:** 10.3390/molecules23051167

**Published:** 2018-05-13

**Authors:** Lihu Zhang, Tingting Wu, Wei Xiao, Zhenzhong Wang, Gang Ding, Linguo Zhao

**Affiliations:** 1 College of Chemical Engineering, Nanjing Forestry University, Nanjing 210037, China; zlh800927@163.com (L.Z.); wttnjfu@163.com (T.W.); 2 Department of Pharmacy, Jiangsu Vocational College of Medicine, Yancheng 224005, Jiangsu, China; 3 Jiangsu Kanion Pharmaceutical Co., Ltd., Lianyungang, Jiangsu 222047, China; xw@kanion.com (W.X.); wzhzh-nj@163.com (Z.W.); dingg2000@126.com (G.D.)

**Keywords:** EGB, TGFs, macroporous resin, anti-inflammation activities

## Abstract

In the present study, the performance and separation characteristics of six macroporous resins for the enrichment and purification of total ginkgo flavonoid *O*-glycosides (TGFs) (quercetin (I), kaempferol (II), isorhamnetin (III)) from Ginkgo Biloba extracts (EGB) are evaluated. The adsorption and desorption properties of TGFs are studied on macroporous resins, including D101, D201, AB-8, HPD400, D301, and D311. Along with the results, AB-8 resin exhibits the best adsorption and desorption capacity for these three ginkgo flavonoid *O*-glycosides among the six resins. Adsorption isotherms are created on AB-8 resin and fit well to the Langmuir (R^2^ > 0.96) and Freundlich (R^2^ > 0.92, 0.3 < 1/n < 0.7) models. After the treatment with gradient elution on AB-8 resin packed chromatography column, the contents of the three main ginkgo flavonoid *O*-glycosides (I, II, and III) increase from 8.93%, 9.88%, and 6.11% in the extracts to 30.12%, 35.21%, and 14.14%, respectively, in the product. The recoveries of compounds I, II, and III are 88.76%, 93.78%, and 60.90%, respectively. Additionally, the anti-inflammatory effects of TGFs are evaluated in LPS-treated RAW 264.7 macrophages, and the result demonstrates that TGFs could significantly inhibit LPS-induced NO release in vitro in a dose-dependent manner compared with the control group. These findings suggest that TGFs could potentially be natural antioxidants and anti-inflammatory ingredients that could be used in pharmaceutical products and functional food additives.

## 1. Introduction

**Figure 1:**
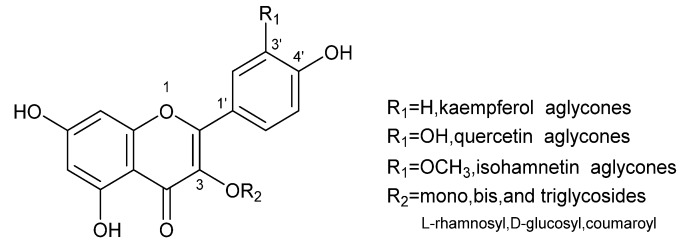
Structural formulas of main three flavonoid *O*-glycosides in EGB.

Ginkgo biloba is called a living fossil and has been surviving on Earth for 180 million years, and EGB has been used for medicinal purposes in China for several thousand years. EGB is available in either a non-standardized form or as a standardized extract containing 24% flavone glycosides, as shown in Figure 1 (e.g., quercetin, kaempferol, isorhamnetin) [1,2], 6% terpene lactones, 7% proanthocyanidins, 13% carboxylic acids and 20% non-flavonol glycosides [3,4]. EGB has gained broad acceptance among the general public, and a broad distribution throughout the health food sector, drug stores, and supermarkets, due to the outstanding body of science supporting its use. 

After extraction, the purity of EGB still did not meet the purity demands, and further purification was carried out. Among all reported technologies [5], macroporous resin adsorption displayed a suitable column separation method owing to its a high adsorption capacity, high efficiency, cheap charge, effortless regeneration and simple procedure [6,7]. These advantages make macroporous resin useful, not only in the field of water treatment, but also in the extraction and purification of bioactive components from the raw extracts of herbal plants [8,9,10].

EGB has been shown to have diverse bioactivities and different pharmacologic actions, such as antioxidation, anti-inflammatory, antitumor and immunomodulatory responses [11]. Thus far, the studies of EGB mainly focus on the treatment of cardiovascular disorders, acute pancreatitis, myocardial disease, vertiginous diseases, obstructive arteriosclerosis and intestinal ischemia reperfusion injury, which are related to inflammatory mediators because of their cheap price and negligible side effects [12,13,14]. Inflammation is the localized response of a tissue to injury, caused by a mechanical or biological agent, or by an abnormal autoimmune response [15]. It has been reported that EGB also possesses anti-inflammatory activity in vitro and in vivo through modulation of proinflammatory cytokines, such as interleukin-6 (IL-6), interleukin-1 beta (IL-1β), cyclooxygenase-2 (COX-2), tumor necrosis factor-alpha (TNF-α), vascular endothelial growth factor (VEGF), inducible nitric oxide synthase (iNOS) and nitric oxide (NO) [16,17]. Thereamong, TGFs are considered primary bioactive ingredients of EGB, although information about the anti-inflammation activity of TGFs from EGB is in short supply.

The object of the present work was to evaluate the adsorption and desorption characteristics of TGFs on different macroporous resins. The physical models of the Langmuir equation and the Freundlich equation, as well as the HPLC method, were used to analyze the kinetic characteristics and separation effects. Based on the information, an efficient method for the preparative separation of TGFs from EGB with an optimal resin. The anti-inflammatory potential effects of TGFs were evaluated in RAW264.7 macrophages.

## 2. Results and Discussion

### 2.1. Screening of Resins for TGFs

According to similarity–intermiscibility theory, total ginkgo flavonoid *O*-glycosides contain non-polar phenyl groups and polar multi-hydroxyl groups. Either non-polar resins or polar macroporous resins are applicable to the adsorption of TGFs. Thus, six macroporous resins ranging from non-polarity to polarity were used for adsorption of TGFs due to the physical and chemical properties, as shown in Table 1.

**Figure 2:**
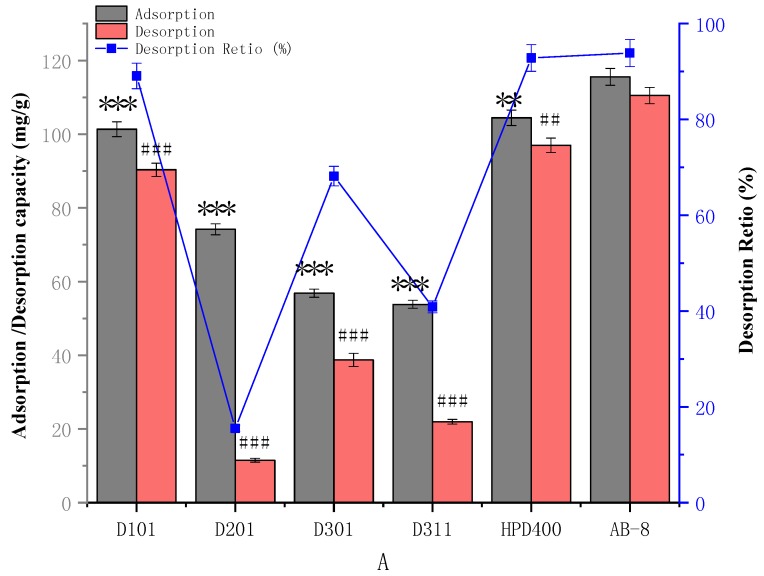
Adsorption, desorption and desorption ratio of TGFs on different resins. Adsorption and desorption capacities are expressed as TGFs content (mg TGFs/g dry resin). The values represent the mean ± SD of the three independent experiments. (** compared with the adsorption capacity for the TGFs of AB-8; ** *p* ≤ 0.01 and *** *p* ≤ 0.001. ## compared with the desorption capacity for the TGFs of AB-8; ## *p* ≤ 0.01 and ### *p* ≤ 0.001.).

Six macroporous resins with distinct physical properties of TGFs from EGB were evaluated and are shown in Figure 2. The adsorption capacity of AB-8 resin towards TGFs was higher than that of HPD400 and D101 resins, while the lower desorption capacity of TGFs on D201, D301 and D311 resins resulted in a lower desorption ratio. However, the desorption ratios of D-101 resins for TGFs were close to those of HPD400 and AB-8 resins, though their adsorption and desorption capacities were lower. In a word, compared with other resins, HPD400 and AB-8 resins possessed better adsorption and desorption capabilities for TGFs. The HPD400 and AB-8 resins exhibited a better adsorption capacity and desorption ratio, not only because of their appropriate polarity, but also because of their greater surface area, which might relate to the capability of the resins and the chemical properties of the adsorbed substance. The adsorption capacities of TGFs on AB-8 or HPD400 resins was 115.57 or 104.46 mg/g dry resin with a desorption ratio of 93.86% or 92.85%, respectively. Therefore, adsorption kinetics experiments were further executed on AB-8 resins.

### 2.2. Adsorption Kinetics

**Figure 3:**
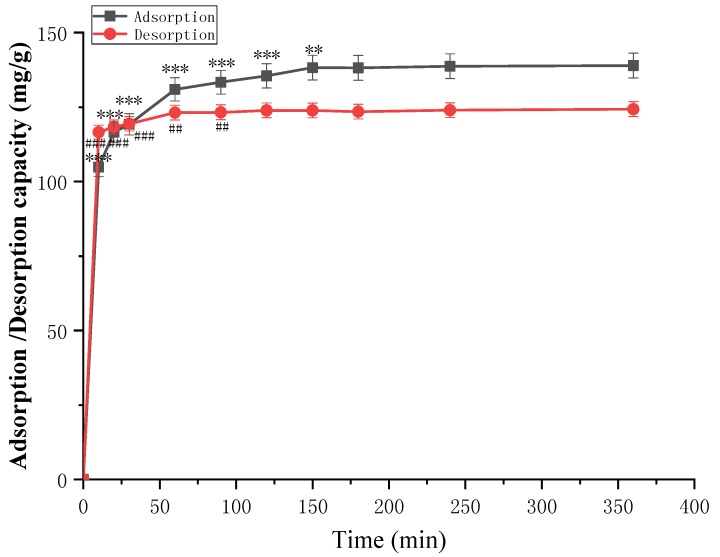
The static adsorption and desorption kinetics of TGFs on AB-8 resin at 30 °C. Adsorption and desorption capacities are expressed as TGFs content (mg TGFs/g dry resin). The values represent the mean ± SD of the three independent experiments. (** compared with the adsorption capacity for the TGFs of 360 min; ** *p* ≤ 0.01 and *** *p* ≤ 0.001. ## compared with the desorption capacity for the TGFs of 360 min; ## *p* ≤ 0.01 and ### *p* ≤ 0.001.).

The kinetics of adsorption that describe the solute uptake rate governing the contact time of the adsorption process is one of the key properties that describes the efficiency of adsorption. Hence, in the present study, the adsorption kinetics of TGFs were determined to understand the adsorption behavior of AB-8 resins. Generally, the adsorption capacity of TGFs increased transiently with adsorption time before reaching adsorption equilibrium. As shown in Figure 3, the equilibrium time for TGFs was 6 h on AB-8 resin. Comparing D101 and HPD400 resins in a comprehensive consideration of the adsorption capacity and desorption ratio (Figure 2), AB-8 resin possessed a great advantage for TGFs. Thus, AB-8 resin was selected as the most suitable resin for the enrichment of TGFs and was used in subsequent tests.

### 2.3. Adsorption Isotherms 

To investigate the adsorption capacity and characterize the adsorption behavior of TGFs, sample solutions with various concentrations of EGB (15.87–37.18 mg/mL) were shaken with AB-8 resin at 25, 30 and 35 °C. Additionally, to interpret the adsorption experimental data, the Langmuir and Freundlich models were used. Table 2 listed the two isotherm equations at different temperatures and two important parameters: Q_m_ value (obtained from the Langmuir isotherm) and 1/n value (obtained from the Freundlich isotherm). The correlations (0.9965–0.9969) for TGFs on AB-8 indicated that the two models were suitable for describing the tested adsorption system in the concentration ranges studied.

Generally, in the Freundlich equation, adsorption was easy to determine when the 1/n value was between 0.1 and 0.7, and it was hard to determine when the 1/n value was beyond 1 [18]. As can be seen from Table 2, the 1/n values were all between 0.1 and 0.7, which shows that the adsorption of TGFs on AB-8 resin happened easily. Thus, the AB-8 resin was conducive to the enrichment of TGFs. Within the ranges of temperatures investigated, the adsorption capacities decreased with the temperature increase, which indicated that the adsorption process was a thermos positive process. Therefore, 30 °C was selected for subsequent experiments.

### 2.4. Effect of pH Value of Sample Solution 

**Figure 4:**
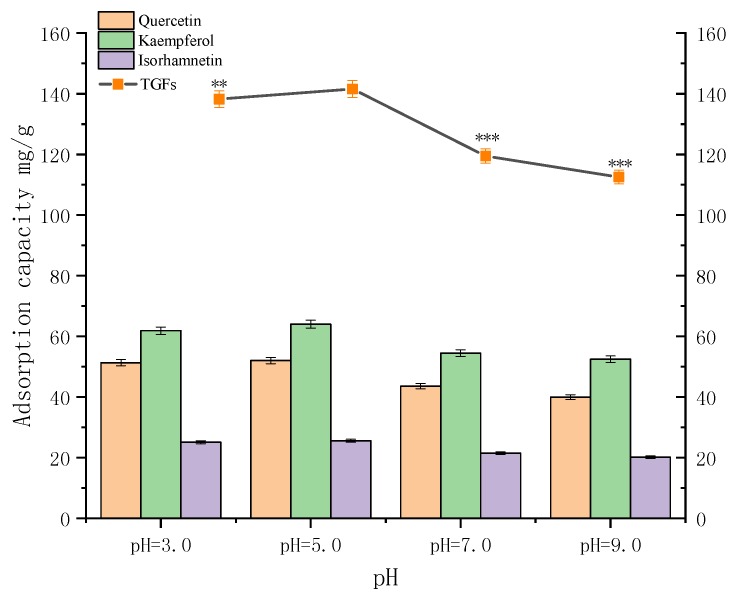
Effects of pH value on adsorption capacity of the three ginkgo flavonoid *O*-glycosides I, II and III. The values represent the mean ± SD of the three independent experiments. (** compared with the adsorption capacity for the TGFs at pH = 5.0; ** *p* ≤ 0.01 and *** *p* ≤ 0.001).

A key parameter affecting the adsorption and desorption capacity of the resins is the pH value of the initial sample solutions. The pH value influences the extent of ionization of solute molecules and therefore affects the affinity between the solutes and solutions [19]. As shown in Figure 4, for AB-8 resin, the adsorption capacity for the TGFs reached its crest value (141.57 mg/g) at pH 5.0, and then decreased gradually with the rise in pH value. The results suggested that hydrogen bonding might play a significant role in the adsorption process on AB-8 resin. At a higher pH value, the hydrogen bonding interactions were reduced, since the phenolic hydroxyl groups in the three ginkgo flavonoid *O*-glycosides hydrolyzed into H^+^ and their corresponding anions, thus resulting in a lower adsorption capacity. Consequently, the pH value of the sample solution was adjusted to 5.0 for subsequent tests [8].

### 2.5. Dynamic Desorption on AB-8 Resin

**Figure 5:**
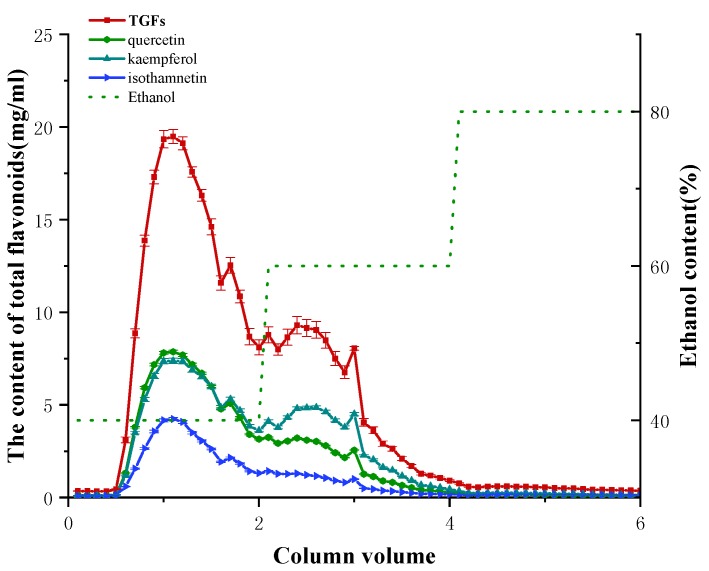
Profile of dynamic desorption of TGFs (I, II, and III) on AB-8 using different ethanol concentrations at 2 BV/h. The values represent the mean ± SD of the three independent experiments.

Dynamic desorption was carried out with a gradient elution mode at a flow rate of 2.0 BV/h. Different elution solvents with the same volume (2 BV) were used to desorb TGFs when the sample loading amount was 2 BV. At 20% ethanol, the TGFs were barely desorbed. When the ethanol concentration was over 20%, the desorption ability increased sharply and achieved a maximum value at 40% ethanol. Thus, a gradient elution procedure with 40%, 60% and 80% ethanol, at a flow rate of 2 BV/h, was used for desorption of TGFs. In addition, the elution volume of each ethanol concentration was modified under the guidance of absorbance. The dried extract of EGB (4.0 g) was dissolved in 20% ethanol. The TGFs were enriched and purified from the sample solution with AB-8 resin. According to the content analysis of TGFs, the elution program was modified as 2 BV of 20% ethanol, 2 BV of 40% ethanol, 2 BV of 60% ethanol and 2 BV of 80% ethanol. As shown in Figure 5, non-adsorption components were washed out by 2 BV of 20% ethanol. Subsequently, some impurities were removed by elution of 2 BV of 20% ethanol. The following 2 BV of 40% ethanol elution made the product rich in TGFs. After separation on AB-8 resin by the gradient elution, the contents of the TGFs reached 79.46%, which was 3.19-fold those in the extract of EGB, and the recovery yields were 92.24%.

Thus, the ultimate separation and purification methodology for TGFs was summarized as follows. Adsorption: Concentrations of TGFs in sample solution 9.64 mg/mL; pH 5.0; flow rate 2 BV/h; temperature 35 °C. Desorption: 20% ethanol and 40% ethanol, each 2 BV; then 2 BV of 60% ethanol; flow rate 2 BV/h.

### 2.6. Anti-Oxidant Activities Analysis

DPPH and ABTS are relatively stable free radical compounds that have been broadly used to test the free radical-scavenging activities of various natural extracts. In the DPPH scavenging assay, the IC_50_ (the concentration required to scavenge 50% of radical) of TGFs, EGB, and ascorbic acid were 38.00 ± 1.06, 47.82 ± 1.05, and 5.73 ± 1.62 μg/mL, respectively. In the ABTS scavenging assay, the IC_50_ values of TGFs, EGB, and ascorbic acid were 24.34 ± 1.12, 42.08 ± 1.01, and 8.06 ± 1.05 μg/mL, respectively.

### 2.7. Anti-Inflammation Activity Analysis

#### 2.7.1. Cytotoxicity Effects of TGFs on Raw 264.7 Cells

**Figure 6:**
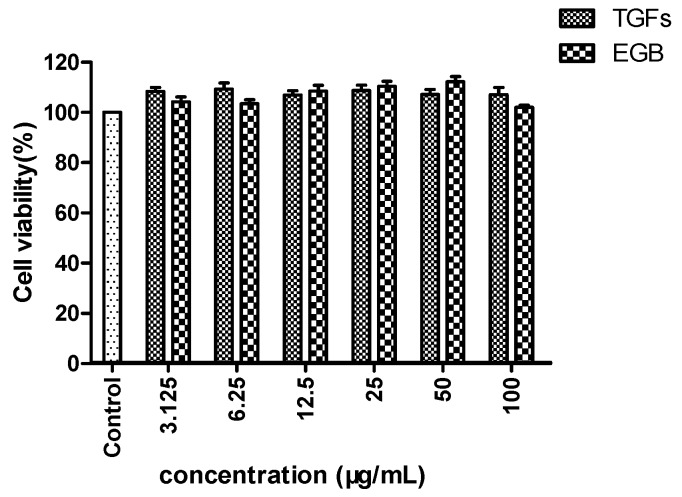
Cell viability detected by MTT assays. The values represent the mean ± SD of the three independent experiments.

To determine the cytotoxicity of TGFs on Raws 264.7 cells, an MTT assay was performed after treatment with LPS and at various concentrations. The results showed that there was no difference among the 3.125, 6.25, 12.5, 25, 50 and 100 µg/mL TGFs and EGB-treated groups (Figure 6), which indicated that cell viability was not significantly affected by up to 100 µg/mL ononin (>95% cell viability). Therefore, TGFs and EGB at concentrations of 3.125, 6.25,12.5, 25, 50 and 100 µg/mL were used in the subsequent experiments. The results showed that cell viabilities (102.0–112.3%) were not significantly (*p* > 0 05) affected by TGFs in the experimental concentration range.

#### 2.7.2. Effects of TGFs on LPS-Treated Overproduction of NO in Raw 264.7 Cells

**Figure 7:**
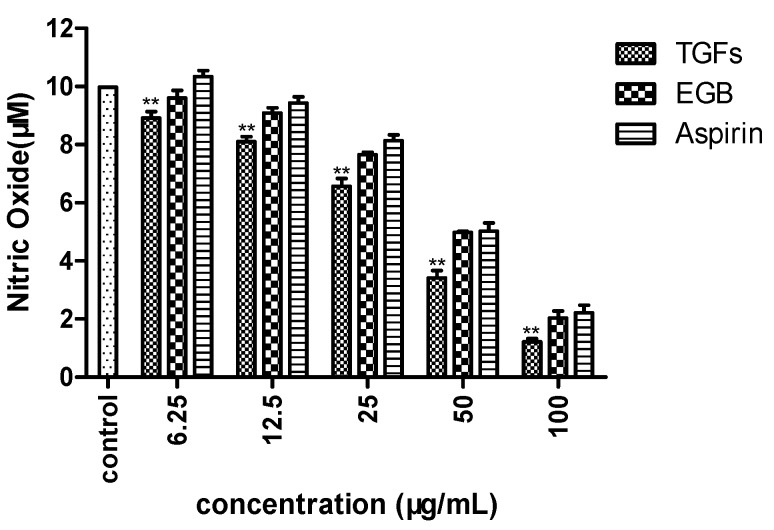
Effects of TGFs, EGB and Aspirin (positive control) on production of NO in RAW 264.7 cells. The NO levels in the cell cultures were measured using Griess assays. The values represent the mean ± SD of the three independent experiments (** compared with control; ** *p* ≤ 0.01).

The anti-inflammatory effects of TGFs and EGB were further evaluated using LPS-treated Raws 264.7 cells. The NO reduction experiment indicated that TGFs and EGB at 50 µg/mL dramatically decreased the NO production of LPS-stimulated Raws 264.7 cells compared to the control group, as shown in Figure 7.

## 3. Experimental Section

### 3.1. Materials and Instruments

EGB was provided by Jiangsu Kanion Pharmaceutical Co., Ltd. (Lianyungang, China); Macroporous resins code AB-8 D101 HPD400 D201 and D301 were purchased from Donghong Chemical Co., Ltd., Xuzhou, Jiangsu, China, Quercetin (I), kaempferol (II), and isorhamnetin (III) standards (98% purity) were purchased from Chengdu Must Biological Technology Co., Ltd. (Chengdu, China). Other chemical regents were analytical grade and obtained from Guoyao Chemical Reagent Co., Ltd. (Shanghai, China). 

B-100 rotary evaporator (Buchi AG, Flawil, Switzerland) was used for concentration of samples, ZHWY200D incubator shaker (Shanghai Zhicheng, Shanghai, China) was used for adsorption and desorption of resins, glass chromatographic columns (30 × 500 mm; 30 × 300 mm) (Nanjing Wanqing Instruments, Nanjing, China) was used for dynamic desorption of AB-8 resin.

### 3.2. Determination of TGFs Content

Samples for TGFs analyses were prepared using the acid hydrolysis method described by Hasler and Sticher [20] with slight changes. Accurately measured samples (500 μL) were transferred to a 2 mL tube, and then 500 μL methanol/25% HCl (4:1) solution was added and the mixture was refluxed at 75 °C for 2 h [21]. The hydrolysis solutions were appropriately diluted with methanol and then filtered through membrane filters (0.22 μm).

$$C_{TGFs}=\left(C_{1}+C_{2}+C_{3}\right)\times 2.51$$

*C_TGFs_*, *C*_1_, *C*_2_ and *C*_3_ were the TGFs, quercetin, kaempferol and isorhamnetin concentration (mg/mL). TGFs were analyzed using HPLC 1200 system (DAD; Agilent, Santa Clara, CA, USA) and a reverse phase C_18_ column (4.6 × 250 mm, 5 μm; Agilent, USA) with a solution system of A (water) and B (methanol). Gradient elution: At 50% B run 0–12 min, 50–80% B 13–15 min, 50% B 15–16 min. The flow ratio was 0.8 mL/min, column temperature was 38 °C, and detection wavelength was 360 nm [22,23]. The contents of hydrolytic aglycones quercetin (I), kaempferol (II) and isorhamnetin (III) were 8.93%, 9.88%, and 6.11%, respectively.

### 3.3. Static Adsorption and Desorption Tests

#### 3.3.1. Adsorption and Desorption Capacities, Desorption Ratio

In order to investigate the static adsorption and desorption properties of these resins, the tests were executed as follows [24]: 5 g (wet weight) resins were put into flasks and 50 mL of sample solution was added (the initial concentration of EGB was 10 mg/mL). The flasks were then shaken (180 rpm) at 30 °C for 8 h. After adsorption equilibrium, the resins were washed with deionized water. Then, the resins were desorbed with 50 mL of 80% ethanol solution, and continually shaken (180 rpm) for 8 h.

The preliminary choices of resins were based on their capacities of adsorption (*Q_e_*)/desorption (*Q_d_*) and desorption ratios (*D*), which were calculated using the following equations [8,25]:

$$Q_{e}=\left(C_{o}-C_{e}\right)\times \frac{V_{i}}{W}$$

$$Q_{d}=\frac{C_{d}\times V_{d}}{W}$$

$$D=\frac{C_{d}\times V_{d}}{\left(C_{o}-C_{e}\right)\times V_{i}}\times 100\%$$

*Q_e_* was the adsorption capacity at adsorption equilibrium (mg/g dry resin). *Q_d_* was the desorption capacity at adsorption equilibrium (mg/g dry resin). *C_o_*, *C_e_,* and *C_d_* were the initial, adsorption equilibrium and desorption concentrations of TGFs (mg/mL). *V_i_* and *V_d_* were the volume of the initial solution and effluent (mL), respectively. W was the weight of dry macroporous resin (g).

The impact of pH on the adsorption capacity of TGFs was studied by mixing 5 g (wet weight) of resin with TGFs solutions (50 mL each) in the pH range of 3.0–9.0. The pH solution was adjusted using concentrated hydrochloric acid or ammonia. Then, the solution was shaken at 30 °C for 8 h in the constant temperature shaking incubator.

#### 3.3.2. Adsorption Isotherms

So as to evaluate the effects of original concentration and temperature on TGFs adsorption, experiments of adsorption isotherm on AB-8 resin were executed. Five groups of 50 mL TGFs solutions with the pH value of 5 at different concentrations were brought into contact with 5 g of wet resins in a constant-temperature shaking incubator at 25, 30 and 35 °C for 8 h. After adsorption equilibrium, TGFs solutions were measured. The Langmuir and Freundlich models, used to evaluate the adsorption behavior, are described in the following equations [26].

Langmuir isotherms:

$$Q_{e}=\frac{Q_{m}\times K_{L}\times C_{e}}{1+K_{L}\times C_{e}}$$

Freundlich isotherms:

Qe=KF×Ce1n

where *K_L_* (mg/mL) was the Langmuir constants, *Q_m_* and 1/n were empirical contants, and *K_F_* was the Freundlich constants, which was an indicator of adsorption capacity [27].

#### 3.3.3. Dynamic Adsorption and Desorption Tests

Dynamic adsorption and desorption tests of AB-8 resin were evaluated using glass columns (500 × 30 mm) with 80 g resin (wet weight). The bed volume (BV) of the resin was 100 mL. After adsorption equilibrium, the column was firstly washed with deionized water, and then eluted with different concentrations of ethanol using the optimized conditions, at a flow rate of 2.0 BV/h. The TGFs effluents were collected every 10 mL for a fraction. The elution volume of each ethanol concentration was adjusted using the HPLC method.

### 3.4. Biological Evaluation

#### 3.4.1. Reagents and Cell Culture

The mouse Raw 264.7 macrophage cell line was obtained from Nanjing University and was cultured in RPMI 1640 medium (Beyotime biotechnology, Shanghai, China) supplemented with 100 U/mL penicillin, 100 mg/mL streptomycin, and 10% heat-inactivated FBS (Beyotime biotechnology) at 37 °C under a humidified air atmosphere of 5% CO_2_. Aspirin was used as a positive control. Compounds were dissolved in dimethyl sulfoxide to make stock solutions and were kept at −20 °C. The nitrate assay kit was purchased from Beyotime biotechnology company. The final concentration of the vehicle in the solution never exceeded 0.1% and had no effects on NO production and cell viability. 2,2′-Azino-bis(3-ethylbenzothiazoline-6-sulfonic acid diammonium salt) (ABTS) and 2,2-diphenyl-1-picrylhydrazyl (DPPH) were purchased from Beyotime biotechnology company.

#### 3.4.2. In Vitro Anti-Oxidant Activity

The DPPH radical scavenging activity of TGFs was measured by the method described by Mishra K [28,29] with modifications. Briefly, a total of 0.1 mL of different concentrations of sample was mixed with 0.9 mL of 0.1 mmol/L DPPH radical ethanol solution. The mixture was shaken and incubated for 30 min at room temperature in the dark, and the absorbance properties were assayed at 517 nm. The IC_50_ values were determined as reported above. The DPPH scavenging effect was calculated as follows:

DPPH radical scavenging rate (%) = [(A_s_ − A_i_)/A_s_] × 100

where A_s_ is the absorbance of DPPH alone, and A_i_ is the absorbance of DPPH in the presence of a specific concentration of TGFs. EGB and ascorbic acid were prepared at the same concentrations and used as reference standards.

The ABTS radical scavenging activity of TGFs was assayed using the method of Dawidowicz and Olszowy [30] with minor modifications. ABTS was produced from the reaction of a 7 mM solution of ABTS with a 2.45 mM solution of potassium persulfate. After the resulting mixture was incubated in the dark at 37 °C for 12–16 h, the ABTS solution was diluted with PBS to yield an absorbance of 0.70 ± 0.02 at 734 nm. TGFs (0.01 mL) of various concentrations were mixed with the ABTS solution (0.19 mL), and the resulting mixtures were allowed to incubate at 37 °C for 30 min. The absorbance properties of the resulting solutions were then measured at 734 nm. The ABTS scavenging effect was calculated based on the following equation:

ABTS radical scavenging rate (%) = [(A_s_ − A_i_)/A_s_] × 100

where A_s_ is the absorbance of ABTS solution, and A_i_ is the absorbance of ABTS solution with sample at different concentrations, respectively. EGB and ascorbic acid were also prepared at the same concentrations and were used as reference standards. The antioxidant activity was expressed as the IC_50_ (mg/mL), which is the TFE concentration that inhibits 50% of the free radicals.

#### 3.4.3. Cell Viability Assay

For analysis of the cytotoxicity of TGFs, Raw 264.7 cells were plated in a 96-well plate at a density of 3 × 10^4^ cells/well, serum-starved for 6 h, and then treated with different concentrations of compounds for 72 h. Next, MTT solution was added to each well, and the cells were incubated for another 4 h at 37 °C. Then, the media were removed, and DMSO was added to dissolve the purple precipitates. The absorbance was measured at a wavelength of 540 nm.

#### 3.4.4. Anti-Inflammatory Assay

Accumulation of nitrite (NO_2_-), an indicator of NO synthase activity, in culture supernatant fluids was measured based on Griess reaction [31]. Briefly, Cells (3 × 10^4^) were seeded in 100 µL of RPMI 1640 into 96-well plates and co-incubated with different concentrations (3.125, 6.25, 12.5, 25, 50, 100 µg/mL) of compounds in the absence or presence of LPS (500 ng/mL) for 48 h. Meanwhile, aspirin was also tested as a positive control. Culture supernatant fluids were mixed with 100 µL Griess reagent at room temperature for 5 min. Using NaNO_2_ to generate a standard curve, nitrite production was measured by an absorbance reading at 540 nm.

### 3.5. Statistical Analysis

All experiments were performed triplicate samples (*n* = 3). The results were expressed as the means ± standard deviation. The experiment data were analyzed statistically by GraphPad Prism 5. The data were subjected to t tests of column analyses. In all statistical analyses, *p* values ≤ 0.05 were regarded as statistically significant and *p* values ≤ 0.01 as very significant.

## 4. Conclusions

In present study, a method for the preparative separation and purification of ginkgo flavonoid *O*-glycosides from EGB with macroporous resin was successfully established. Among the six typical macroporous resins investigated, AB-8 resin was selected due to its better adsorption capacity and ratio of desorption. The equilibrium adsorption experimental data on AB-8 resin of I, II, and III were well suited to the Langmuir and Freundlich isotherms. Further dynamic adsorption/desorption experiments on AB-8 column were conducted to obtain the optimal parameters. The contents of the flavonoid *O*-glycosides (I, II, and III) in the EGB sharply increased from 8.93%, 9.88%, and 6.11% to 30.12%, 35.21%, and 14.14% in the TGFs. The recovery rate of compounds I, II, and III were 88.76%, 93.78%, and 60.90%. The results verified a good adsorption and desorption chromatography for the preparative enrichment and separation flavonoid *O*-glycosides from medical herbs. These results of the anti-inflammatory effect evaluation of TGFs showed it could significantly inhibit LPS-induced NO release in vitro compared with the control group in a dose-dependent fashion. This study offers a theoretical basis for further study on bioactive flavonoid *O*-glycosides from EGB, which shows potential for functional food and pharmaceutical applications in industry.

## Figures and Tables

**Table 1:** Physical and chemical properties of microporous adsorption resins.

Trade Name	Surface Area (m^2^/g)	Average Pore Diameter (nm)	Particle Diameter (mm)	Bulk Density (g/mL)	Type	Moisture Content (%)
D101	500–550	9–11	0.30–1.25	0.80–0.85	No-polar	66.61
D201	≥650	15–20	0.30–1.25	0.65–0.75	Strong-base anion	49.43
D301	350–400	15–20	0.30–1.25	0.65–0.72	Weak-base anion	52.52
D311	350–430	15–20	0.32–1.25	0.70–0.80	Weak-base anion	47.33
AB-8	450–520	13–14	0.30–1.25	0.65–0.75	Weak-polar	64.46
HPD400	≥650	10–15	0.30–1.25	0.65–0.70	Polar	58.24

**Table 2:** Langmuir and Freundlich parameters of I, II, III and TGFs on AB-8 resin.

	**T(°C)**	**Langmuir Equation**	**R^2^**
quercetin	25 °C	Q_e_ = (146.67C_e_)/(8.04 + C_e_)	0.9974
	30 °C	Q_e_ = (132.14C_e_)/(7.61 + C_e_)	0.9998
	35 °C	Q_e_ = (141.43C_e_)/(6.52 + C_e_)	0.9991
kaempferol	25 °C	Q_e_ = (205.73C_e_)/(8.43 + C_e_)	0.9946
	30 °C	Q_e_ = (170.32C_e_)/(6.97 + C_e_)	0.9923
	35 °C	Q_e_ = (184.15C_e_)/(6.05 + C_e_)	0.9899
isorhamnetin	25 °C	Q_e_ = (118.65C_e_)/(9.78 + C_e_)	0.9916
	30 °C	Q_e_ = (77.90C_e_)/(6.46 + C_e_)	0.9900
	35 °C	Q_e_ = (94.99C_e_)/(6.00 + C_e_)	0.9750
TGFs	25 °C	Q_e_ = (462.73C_e_)/(24.97 + C_e_)	0.9936
	30 °C	Q_e_ = (383.81C_e_)/(21.39 + C_e_)	0.9903
	35 °C	Q_e_ = (433.71C_e_)/(18.98 + C_e_)	0.9637
	**T(°C)**	**Freundlich Equation**	**R^2^**
quercetin	25 °C	Q_e_ = 26.55C_e_^0.472^	0.9974
	30 °C	Q_e_ = 31.73C_e_^0.358^	0.9820
	35 °C	Q_e_ = 28.90C_e_^0.491^	0.9861
kaempferol	25 °C	Q_e_ = 32.53C_e_^0.526^	0.9946
	30 °C	Q_e_ = 39.33C_e_^0.386^	0.9895
	35 °C	Q_e_ = 35.50C_e_^0.514^	0.9651
isorhamnetin	25 °C	Q_e_ = 13.62C_e_^0.660^	0.9946
	30 °C	Q_e_ = 16.34C_e_^0.449^	0.9750
	35 °C	Q_e_ = 16.22C_e_^0.600^	0.9563
TGFs	25 °C	Q_e_ = 39.10C_e_^0.545^	0.9978
	30 °C	Q_e_ = 55.89C_e_^0.395^	0.9871
	35 °C	Q_e_ = 43.66C_e_^0.536^	0.9280

## References

[1] Jager A.K., Saaby L. (2011). Flavonoids and the CNS. Molecules.

[2] Suciu S., Olah N.K., Morgovan C., Toma C.C., Dărăban A. (2013). Pharmacokinetic Drug-Herbal Dietary Supplements Interactions Clinically Significant.

[3] Singh B., Kaur P., Gopichand, Singh R.D., Ahuja P.S. (2008). Biology and chemistry of Ginkgo biloba. Fitoterapia.

[4] van Beek T.A., Montoro P. (2009). Chemical analysis and quality control of Ginkgo biloba leaves, extracts, and phytopharmaceuticals. J. Chromatogr. A.

[5] Zhang J., Hayat K., Zhang X., Tong J., Xia S. (2010). Separation and Purification of Flavonoid from Ginkgo Extract by Polyamide Resin. Sep. Sci. Technol..

[6] Sun Y., Yuan H., Hao L., Min C., Cai J., Liu J., Cai P., Yang S. (2013). Enrichment and antioxidant properties of flavone C-glycosides from trollflowers using macroporous resin. Food Chem..

[7] Vien L.T., Van Q.T.T., Hanh T.T.H., Huong P.T.T., Thuy N.T.K., Cuong N.T., Dang N.H., Thanh N.V., Cuong N.X., Nam N.H. (2017). Flavonoid glycosides from Barringtonia acutangula. Bioorg. Med. Chem. Lett..

[8] Du H., Wang H., Yu J., Liang C., Ye W., Li P. (2012). Enrichment and purification of total flavonoidC-glycosides from Abrus mollis extracts with macroporous resins. Ind. Eng. Chem. Res..

[9] Wan P., Sheng Z., Han Q., Zhao Y., Cheng G., Li Y. (2014). Enrichment and purification of total flavonoids from Flos Populi extracts with macroporous resins and evaluation of antioxidant activities in vitro. J. Chromatogr. B.

[10] Xie Y., Guo Q.S., Wang G.S. (2016). Preparative separation and purification of the total flavonoids in Scorzonera austriaca with Macroporous Resins. Molecules.

[11] Lu C.-H., Hwang C.-W., Chen N.-F., Liu W.-S., Hsiao Y.-F., Wu W.-T. (2012). In vivo effects of Ginkgo biloba extract on interleukin-6 cytokine levels in patients with neurological disorders. Indian J. Pharmacol..

[12] Maitra I., Marcocci L., Droy-Lefaix M.T., Packer L. (1995). Peroxyl radical scavenging activity of Ginkgo biloba extract EGb 761. Biochem. Pharmacol..

[13] Zeybek N., Gorgulu S., Yagci G., Serdar M., Simsek A., Kaymakcioglu N., Deveci S., Ozcelik H., Tufan T. (2003). The effects of gingko biloba extract (EGb 761) on experimental acute pancreatitis. J. Surg. Res..

[14] Yoshikawa T., Naito Y., Kondo M. (1999). Ginkgo biloba leaf extract: Review of biological actions and clinical applications. Antioxid. Redox Sign..

[15] Ilic N.M., Dey M., Poulev A.A., Logendra S., Kuhn P.E., Raskin I. (2014). Anti-inflammatory activity of grains of paradise (Aframomum melegueta Schum) extract. J. Agric. Food Chem..

[16] Wadsworth T.L., Mcdonald T.L., Koop D.R. (2001). Effects of Ginkgo biloba extract (EGb 761) and quercetin on lipopolysaccharide-induced signaling pathways involved in the release of tumor necrosis factor-alpha. Biochem. Pharmacol..

[17] Biddlestone L., Corbett A.D., Dolan S. (2007). Oral administration of Ginkgo biloba extract, EGb-761 inhibits thermal hyperalgesia in rodent models of inflammatory and post-surgical pain. Br. J. Pharmacol..

[18] Zhu Y.X., Song H., Zhang X., Chen C.Y., Zhao S.L., Ge F., Liu D.Q. (2017). Recovery of Flavonoids from walnuts de-pellicle wastewater with macroporous resins and evaluation of antioxidant activities in vitro. J. Food Process. Eng..

[19] Zhao P., Qi C., Wang G., Dai X.P., Hou X.H. (2015). Enrichment and purification of total flavonoids from cortex juglandis mandshuricae extracts and their suppressive effect on carbon tetrachloride-induced hepatic injury in Mice. J. Chromatogr. B.

[20] Hasler A., Gross G.A., Meier B., Sticher O. (1992). Complex flavonol glycosides from the leaves of Ginkgo biloba. Phytochemistry.

[21] Commission C.P. (2015). Pharmacopoeia of the People’s Republic of China.

[22] Lin G., Chen A., Pei J., Zhao L., Fang X., Gang D., Wang Z., Wei X., Feng T. (2017). Enhancing the thermostability of α-L-rhamnosidase from aspergillus terreus and the enzymatic conversion of rutin to isoquercitrin by adding sorbitol. BMC Biotechnol..

[23] Zhang X., Pei J., Zhao L., Tang F., Fang X., Xie J. (2016). Overexpression and characterization of CCD4 from Osmanthus fragrans and β-ionone biosynthesis from β-carotene in vitro. J. Mol. Catal. B-Enzym..

[24] Sandhu A.K., Gu L.W. (2013). Adsorption/Desorption Characteristics and Separation of Anthocyanins from Muscadine (Vitis rotundifolia) Juice Pomace by Use of Macroporous Adsorbent Resins. J. Agric. Food Chem..

[25] Du Z.Q., Wang K., Tao Y., Chen L.X., Qiu F. (2012). Purification of baicalin and wogonoside from Scutellaria baicalensis extracts by macroporous resin adsorption chromatography. J. Chromatogr. B.

[26] Kim J., Yoon M., Yang H., Jo J., Han D., Jeon Y.J., Cho S. (2014). Enrichment and purification of marine polyphenol phlorotannins using macroporous adsorption resins. Food Chem..

[27] Ren J., Zheng Y., Lin Z., Han X., Liao W. (2017). Macroporous resin purification and characterization of flavonoids from *Platycladus orientalis* (L.) Franco and their effects on macrophage inflammatory response. Food Funct..

[28] Mishra K. (2012). Estimation of antiradical properties of antioxidants using DPPH assay: A critical review and results. Food Chem..

[29] Ping J., Huafang C., Haibin H., Xinye B., Lan X. (2017). Ultrasonic-assisted extraction of total flavonoids from branches and leaves of Taxus cuspidata Sieb. et Zucc. and its antioxidant activity. Chem. Ind. For. Prod..

[30] Dawidowicz L., Olszowy M. (2011). Antioxidant properties of BHT estimated by ABTS assay in systems differing in pH or metal ion or water concentration. Eur. Food Res. Technol..

[31] Ping J., Jia X., Fei W., Mary H., Grace M., Ann L., Rui X. (2017). α-Amylase and α-Glucosidase inhibitory activities of phenolic extracts from *eucalyptus grandis* × *E. urophylla* Bark. J. Chem..

